# Phase-specific changes in anthropometric and physical fitness outcomes among Chinese upper-secondary students before, during, and after the COVID-19 pandemic: the moderating role of educational track

**DOI:** 10.3389/fpubh.2026.1771290

**Published:** 2026-03-04

**Authors:** Chenlu Xu, Chendong Xu, Shenxing Du

**Affiliations:** 1 Zhejiang Pan'an High School, Jinhua, Zhejiang, China; 2 Department of Rehabilitation, Dongyang Red Cross Hospital, Jinhua, Zhejiang, China

**Keywords:** adolescent physical fitness, COVID-19 pandemic, educational stratification, health inequality, school type, school-based surveillance

## Abstract

**Background:**

The COVID-19 pandemic substantially disrupted school-based physical activity worldwide; however, how such disruptions differentially affect distinct domains of adolescent physical fitness across educational tracks remains insufficiently understood.

**Methods:**

Using large-scale school-based fitness surveillance data collected before, during, and after the COVID-19 pandemic, we examined phase-specific changes in anthropometric indicators and physical fitness outcomes among Chinese upper secondary school students aged 15–18 years. Students were stratified by educational track (general academic vs. vocational education), and outcomes spanning explosive power, endurance, flexibility, and anthropometric measures were analyzed to assess phase effects and phase-by-school-type interactions.

**Results:**

Statistically robust phase-related variations and phase-by-school-type interactions were observed across all fitness domains, with highly domain-specific responses. Anthropometric indicators followed overall upward trajectories consistent with long-term secular patterns, although students in vocational education exhibited greater temporal sensitivity to pandemic-related disruption. Explosive power showed relatively small phase effects but large and persistent between-school differences, whereas endurance and flexibility displayed pronounced phase-dependent changes, including delayed differentiation in endurance performance and a temporary narrowing of between-school disparities in flexibility during the pandemic.

**Conclusions:**

These findings demonstrate that large-scale societal disruptions, such as the COVID-19 pandemic, can alter adolescent physical fitness in domain- and context-specific ways, reshaping developmental patterns and inequality dynamics rather than uniformly depressing fitness levels. The educational track plays a critical role in moderating vulnerability and recovery across fitness domains, underscoring the need for fitness monitoring and intervention strategies that are sensitive to both domain-specific characteristics and the educational context.

## Introduction

The COVID-19 pandemic represented an unprecedented global public health disruption, substantially altering daily routines, movement behaviors, and health-related practices worldwide (1–3). Population-level evidence consistently documents marked reductions in physical activity, increases in sedentary behavior, and widespread interruptions to structured exercise opportunities during periods of lockdown and social restriction, particularly in East Asia, where school closures and mobility controls were often prolonged (4–6). Adolescents constitute a population of particular public health concern, as this developmental stage is characterized by rapid physical growth, consolidation of behavioral habits, and heightened sensitivity to environmental constraints (7, 8). Importantly, disruptions occurring during adolescence may exert lasting influences on health trajectories beyond the immediate pandemic period (9) and into young adulthood. Against this background, examining phase-specific changes in physical fitness among high school-aged (upper-secondary) adolescents aged 15–18 years provides critical insight into the population-level health impact of pandemic-related restrictions and informs strategies for post-pandemic recovery in this population.

Within this broader health context, physical fitness represents a particularly informative indicator of adolescent health. As an integrative construct encompassing cardiovascular, respiratory, musculoskeletal, and metabolic capacities, adolescent physical fitness reflects both functional health status and longer-term health prospects across the life course (10). Unlike many self-reported health behaviors, physical fitness can be objectively assessed through standardized school-based testing, making it particularly well-suited for population surveillance and policy-relevant evaluation and monitoring (11). In China, large-scale fitness monitoring embedded in the Chinese National Survey on Students' Constitution and Health (CNSSCH) has generated near-census data, providing a unique opportunity to examine long-term shifts in youth growth and physical fitness patterns (12, 13). Analyses of successive national surveys have revealed substantial changes in anthropometric indicators since the mid-1990s, accompanied by declines in multiple fitness components, with endurance- and respiratory-related outcomes showing particular vulnerability (11, 13).

Accumulating evidence suggests that the COVID-19 pandemic was associated with unfavorable changes in youth physical fitness, including increases in body mass index and deterioration in cardiorespiratory fitness and flexibility (14–16). However, two important gaps remain in the literature. First, most studies focus on pre-and during contrasts and lack a clearly defined post-pandemic phase, limiting insights into recovery dynamics. Second, adolescents are often treated as a homogeneous population, with limited attention paid to structural heterogeneity within the education system. In China, these limitations are particularly significant. Upper-secondary education is organized through a strong academic–vocational tracking system following the high school entrance examination (Zhongkao), which is associated with systematic differences in institutional resources, curriculum structure, and daily activity environments (17, 18). Beyond academic outcomes, emerging evidence suggests that vocational-track students differ from their general academic peers regarding health-related behaviors and opportunities for physical activity (16, 19, 20). These structural differences raise the possibility that pandemic-related disruptions did not affect all adolescents uniformly, but instead interacted with educational stratification to shape divergent patterns of fitness change and recovery. Accordingly, the present study aimed to characterize phase-specific changes in anthropometric indicators and multiple domains of physical fitness among Chinese upper-secondary students before, during, and after the COVID-19 pandemic, and to examine whether these patterns differed systematically between general academic and vocational education tracks within the same surveillance system. Leveraging large-scale school-based fitness surveillance data collected from 2016 to 2024, this analysis provides a structured assessment of overall population-level shifts and school-type-specific recovery dynamics following pandemic-related disruption.

## Methods

### Study design and data source

This study is a retrospective secondary analysis of routine school-based physical fitness surveillance data collected between 2016 and 2024 under the supervision of the local education bureau. The monitoring system adheres to the national Student Physical Fitness and Health Standards of China and is embedded within the framework of the CNSSCH (12). Consistent with prior large-scale analyses based on successive CNSSCH survey waves (12, 13) and recent provincial surveillance conducted during the COVID-19 period (21), the present dataset was derived from census-style, school-organized testing implemented as part of routine educational administration rather than convenience sampling. Importantly, assessments were administered by school physical education teachers following standardized protocols; in line with CNSSCH-related field practice, testing personnel are typically trained and required to pass measurement examinations, with on-site supervision to support quality control and minimize measurement error (21, 22).

All raw records were processed by the education bureau prior to release to the research team. Direct personal identifiers (e.g., name, student ID, home address) were removed, and each record was assigned a study-specific pseudonymous identifier. The re-identification/linkage key was retained exclusively by the data holder under restricted access and was not available to the investigators, consistent with established best practices for privacy-preserving secondary use of educational and health data. As a result, the analytic dataset contained no information that would permit re-identification of individual students by the research team. Although the study involved only fully anonymized secondary data with no direct participant contact, the research protocol was submitted for institutional ethics review. The Institutional Review Board determined that the project met criteria for exemption from full review, as it involved anonymized records.

The analytic dataset comprised upper-secondary school students from two cities in China and included two educational tracks: general academic senior high schools (hereafter, general high schools) and secondary vocational or technical schools (hereafter, vocational high schools). In China, students complete nine years of compulsory education and subsequently sit for the high school entrance examination (Zhongkao), which serves as the primary mechanism for allocation into upper-secondary educational tracks (17, 18).

To examine the pandemic-related changes, calendar years were grouped into three mutually exclusive phases: pre-pandemic (2016–2019), during-pandemic (2020–2022), and post-pandemic (2023–2024). The phase-specific sample sizes are presented in Table 1. This study used repeated cross-sectional surveillance data; the same individuals were not followed up across years. Accordingly, associations across pandemic phases reflect population-level temporal patterns rather than within-person developmental trajectories, and causal inferences about individual adaptation or recovery are not warranted.

**Table 1:** Sample characteristics by pandemic phase and school type.

**Phase**	**School type**	***N***	**Male (%)**	**Age (years)**
Pre	Regular schools	14,757	43.9	16.37 ± 0.95
	Vocational schools	9,735	66.3	16.19 ± 0.96
During	Regular schools	7,085	45.8	16.39 ± 0.95
	Vocational schools	6,563	62.8	16.15 ± 0.94
Post	Regular schools	10,642	49.5	16.41 ± 0.96
	Vocational schools	10,656	61.3	16.27 ± 0.98

Values are presented as mean ± standard deviation unless otherwise indicated. Age was calculated as the difference between the measurement year and birth year.

### Physical fitness measures

Annual assessments followed standardized national protocols and included a battery of tests broadly consistent with international recommendations for population-based fitness surveillance (10, 11). The following indicators were included:

- Height (cm) and body mass (kg) were measured during routine examinations. Body mass index (BMI, kg/m^2^) was calculated as body mass divided by height squared.
- Forced vital capacity (mL) was assessed using spirometry in accordance with national testing standards and is commonly used as an indicator of respiratory function in Chinese school-based surveillance.
- 50-m sprint speed (m/s) was derived as distance divided by time; higher values indicate better performance.
- Standing long jump (cm) assessed lower-limb explosive power; the best attempt was recorded.
- Sit-and-reach (cm) measured flexibility using a standardized apparatus.
- Endurance running speed (m/s) was calculated from sex-specific distances (800 m for girls; 1,000 m for boys) to provide a directionally consistent indicator of cardiorespiratory endurance performance. (11).

### Data processing and variable derivation

A standardized preprocessing pipeline was applied prior to inferential analyses. Records were restricted to the three predefined pandemic phases and to upper-secondary grades. For each outcome, observations with missing values for that outcome were excluded (available-case analysis). As a result, analytic sample sizes vary slightly by outcome; detailed counts are provided in Supplementary material. BMI was computed from cleaned height and weight values. Sprint speed and endurance running speed were derived as described above so that higher values consistently reflected better performance across all fitness outcomes. Consistent with the nature of routine administrative surveillance data, no additional outlier trimming was applied beyond the handling of missing values, as implausible or invalid measurements were recorded as missing at the data collection stage. Age (years) was computed as measurement year minus birth year and was used for descriptive reporting only. Age was not included as a covariate in the primary models because mean age was highly comparable across pandemic phases within each school type (Table 1). After preprocessing, 59,438 observations remained in the primary analytic cohort used for descriptive and inferential analyses.

### Statistical analysis

All statistical analyses were conducted using Python (version 3.11; Python Software Foundation, Wilmington, DE, United States). Data manipulation and pre-processing were performed with the pandas (open-source, pandas development team) and numpy (open-source, NumPy developers) libraries. Regression analyses were implemented using the statsmodels (open-source, statsmodels development team) package. Figures were generated using matplotlib (open-source, Matplotlib development team) and seaborn (open-source, seaborn contributors).

Descriptive statistics are presented as mean ± standard deviation. Sample characteristics by pandemic phase and school type are summarized in Table 1, and full descriptive statistics for all outcomes are provided in Supplementary Table S1. To quantify differences in anthropometric and physical fitness outcomes, for each outcome we fit a single pre-specified pooled ordinary least squares (OLS) linear model including pandemic phase (pre-, during-, post-pandemic), school type (general academic vs. vocational), their interaction. For each outcome, we specified pandemic phase, school type, and their interaction as predictors:


$$Y=\beta_{0}+\beta_{1}\text{Phase}+\beta_{2}\text{SchoolType}+\beta_{3}\text{(Phase}\times \text{SchoolType)}+\varepsilon .$$

This pooled model was used to formally evaluate main effects of pandemic phase and school type as well as the Phase × SchoolType interaction across the full sample. Omnibus tests were conducted using F statistics to assess overall main effects and interaction effects prior to planned contrasts (23, 24).

This factorial linear-model framework (OLS) is widely used for large-scale surveillance data to estimate main and interaction effects (Phase, SchoolType, and Phase × SchoolType) and to derive model-based contrasts in a transparent and reproducible manner (25, 26). Planned contrasts included (i) phase-wise comparisons within each school type and (ii) school-type differences within each pandemic phase. Results for (i) are reported in Supplementary Table S2 and results for (ii) in Supplementary Table S3. For planned contrasts, *p* values were adjusted for multiplicity using the Holm method within each outcome. For regression-based contrasts, effect magnitude was quantified using adjusted mean differences with corresponding confidence intervals. For complementary omnibus analyses reported in the main text, effect sizes are summarized using partial eta-squared ($\eta_{p}^{2}$), with values of approximately 0.01, 0.06, and 0.14 interpreted as small, moderate, and large effects, respectively (27). All statistical tests were two-sided, with statistical significance defined as *p* < 0.05.

## Results

### Main and interaction effects of pandemic phase and school type

To assess the overall associations of pandemic phase and school type with anthropometric and physical fitness outcomes, linear models including pandemic phase, school type, and their interaction were fitted for each outcome. Omnibus tests for main and interaction effects are summarized in Table 2.

**Table 2:** Omnibus F tests from ordinary least squares (OLS) models for the main and interaction effects of pandemic phase and school type on anthropometric and physical fitness outcomes.

**Outcome**	**Effect**	**df**	***F***	***p* value**	** $\eta_{p}^{2}$ **
Height	Phase	2	538.46	< 0.001	0.018
	School type	1	573.55	< 0.001	0.010
	Phase × School type	2	277.00	< 0.001	0.009
Weight	Phase	2	457.21	< 0.001	0.015
	School type	1	20.15	< 0.001	< 0.001
	Phase × School type	2	74.15	< 0.001	0.002
BMI	Phase	2	226.39	< 0.001	0.008
	School type	1	34.53	< 0.001	< 0.001
	Phase × School type	2	31.66	< 0.001	0.001
Sprint speed	Phase	2	225.97	< 0.001	0.008
	School type	1	2795.22	< 0.001	0.045
	Phase × School type	2	51.40	< 0.001	0.002
Long jump	Phase	2	376.22	< 0.001	0.013
	School type	1	3610.66	< 0.001	0.057
	Phase × School type	2	56.41	< 0.001	0.002
Forced vital capacity	Phase	2	92.08	< 0.001	0.003
	School type	1	5108.05	< 0.001	0.079
	Phase × School type	2	110.20	< 0.001	0.004
Endurance speed	Phase	2	11.86	< 0.001	< 0.001
	School type	1	6703.12	< 0.001	0.101
	Phase × School type	2	68.87	< 0.001	0.002
Sit-and-reach	Phase	2	745.69	< 0.001	0.024
	School type	1	3201.59	< 0.001	0.051
	Phase × School type	2	397.07	< 0.001	0.013

Omnibus *F* tests were derived from linear models including pandemic phase, school type, and their interaction. $\eta_{p}^{2}$ denotes partial eta-squared.

Across all outcomes, pandemic phase was significantly associated with anthropometric and physical fitness measures (all *p* < 0.001), indicating systematic temporal variation across the pre-pandemic, during-pandemic, and post-pandemic periods. School type was also significantly associated with all outcomes. For anthropometric measures (height, weight, and body mass index), the magnitude of school-type differences was small (partial $\eta_{p}^{2}<0.02$), suggesting modest overall disparities between general academic and vocational school students.

In contrast, substantially larger school-type associations were observed for physical fitness outcomes. The strongest associations were found for endurance running speed ($\eta_{p}^{2}=0.101$), Forced vital capacity ($\eta_{p}^{2}=0.079$), standing long jump performance ($\eta_{p}^{2}=0.057$), and sit-and-reach flexibility ($\eta_{p}^{2}=0.051$), indicating pronounced differences between students from general academic and vocational education tracks across multiple fitness domains.

**Figure 1:**
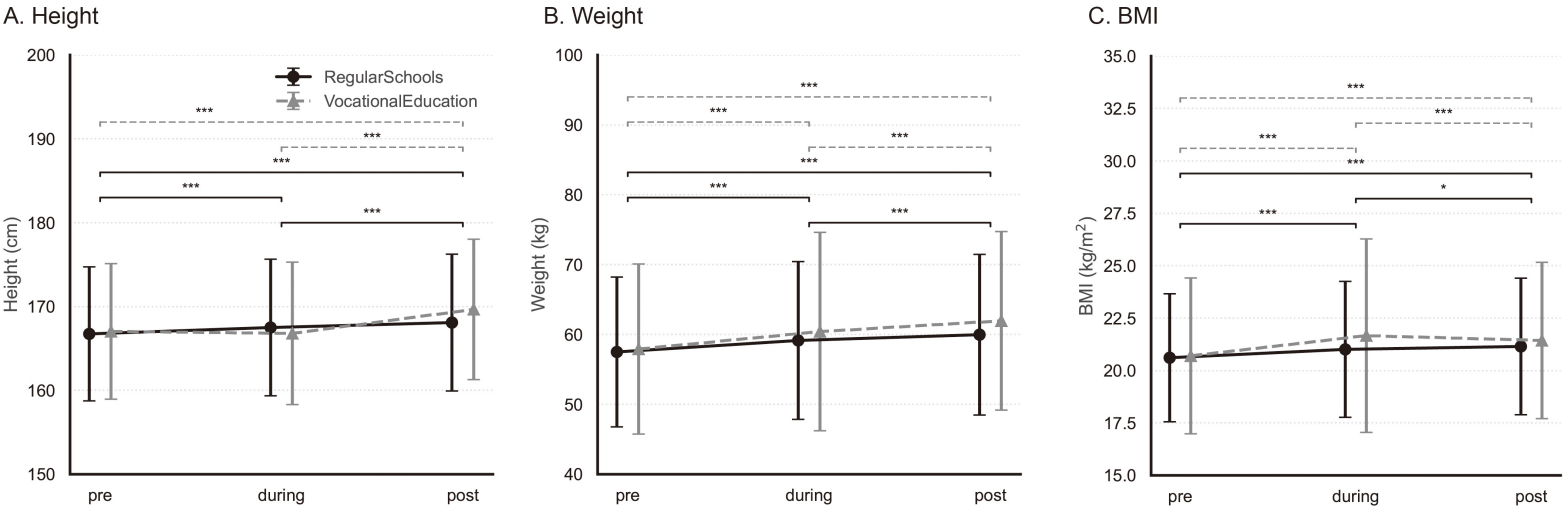
Anthropometric changes across pandemic phases by school type. Changes in anthropometric measures across pandemic phases for RegularSchools and VocationalEducation: **(A)** height, **(B)** weight, and **(C)** body mass index (BMI). Points and lines show the raw sample mean, and error bars indicate ±1 SD. Phase-wise pairwise comparisons within each school type were tested using planned contrasts from the pooled OLS model including Phase, School type, and their interaction (Phase × School type), with Holm-adjusted *p* values; statistically significant contrasts are indicated by horizontal bars. Solid black lines represent Regular Schools, and dashed gray lines represent Vocational Education. ^*^ indicates Holm-adjusted *p* < 0.05 and ^***^ indicates Holm-adjusted *p* < 0.001.

**Figure 2:**
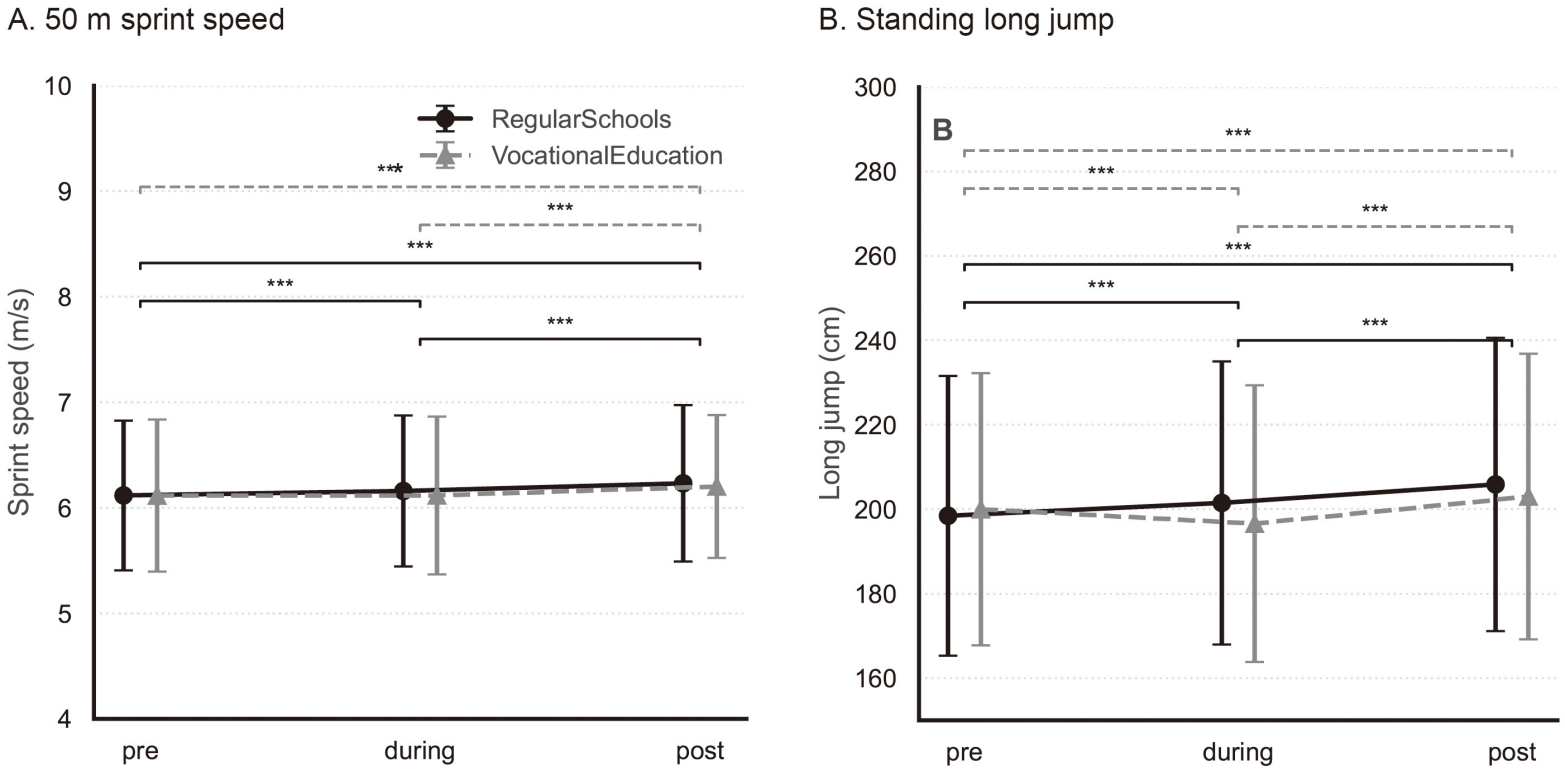
Changes in strength- and power-related fitness outcomes. Changes in physical fitness outcomes across pandemic phases by school type: **(A)** 50 m sprint speed, **(B)** standing long jump. Points and lines show the raw sample mean, and error bars indicate ±1 SD. Solid black lines indicate RegularSchools, and dashed gray lines indicate VocationalEducation. Horizontal bars denote statistically significant phase-wise planned contrasts within school types from the pooled OLS model including Phase, SchoolType, and their interaction (Phase × SchoolType), using Holm-adjusted *p* values (^*^p < 0.05; ^**^p < 0.01).

**Figure 3:**
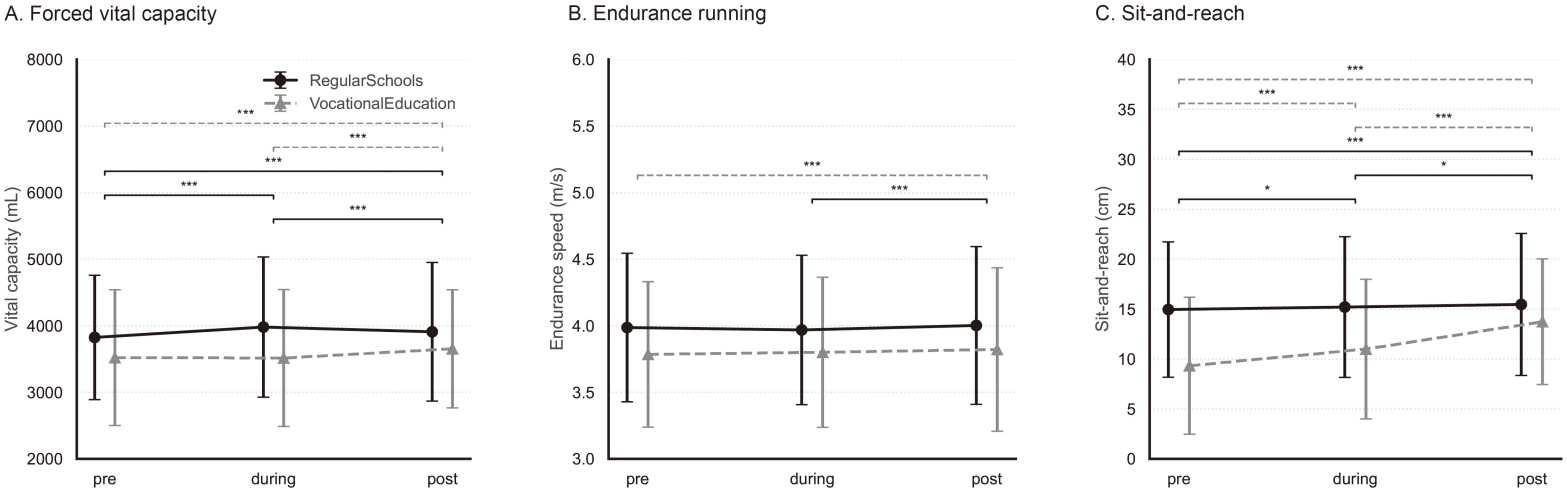
Changes in endurance- and flexibility-related fitness outcomes. Changes in physical fitness outcomes across pandemic phases by school type: **(A)** Forced vital capacity, **(B)** endurance running speed, and **(C)** sit-and-reach performance. Points and lines show the raw sample mean, and error bars indicate ±1 SD. Solid black lines indicate Regular schools, and dashed gray lines indicate Vocational education. Horizontal bars denote statistically significant phase-wise planned contrasts within school types from the pooled OLS model including Phase, School type, and their interaction (Phase × School type), using Holm-adjusted *p* values (^*^p < 0.05; ^**^p < 0.01; ^***^p < 0.001).

Significant interaction effects between pandemic phase and school type were identified for all outcomes (all *p* < 0.001), although the associated effect sizes were consistently small (partial $\eta_{p}^{2}\leq 0.013$). These interactions indicate that the magnitude and direction of school-type differences varied across pandemic phases, thereby justifying subsequent phase-specific and school-type–stratified contrasts presented in the following sections and illustrated in Figures 1–3.

### Strength- and power-related fitness outcomes

Strength- and power-related fitness outcomes, assessed using 50-m sprint speed and standing long jump performance, showed clear phase-related variation and substantial between-track differences (Figure 2). Omnibus OLS tests indicated significant main effects of pandemic phase for both outcomes (all *p* < 0.001; Table 2).

Sprint speed increased from pre- to post-pandemic in both school types. In general academic schools, improvements were observed across all consecutive phases (all Holm-adjusted *p* < 0.001), whereas in vocational schools the pre- to during-pandemic change was not significant (Holm-adjusted *p* = 0.99) and the improvement occurred primarily from during- to post-pandemic (Holm-adjusted *p* < 0.001) (Supplementary Table S2; Figure 2A). A pronounced main effect of school type was observed (*F* = 2795.22, *p* < 0.001; partial $\eta_{p}^{2}=0.045$), indicating consistently higher performance among students from general academic schools across all phases. Although the pandemic phase × school-type interaction was statistically significant, its magnitude was small (partial $\eta_{p}^{2}=0.002$), suggesting broadly similar temporal patterns between school types.

Standing long jump performance increased monotonically across phases in general academic schools (all Holm-adjusted *p* < 0.001). In vocational schools, performance decreased from pre- to during-pandemic (Holm-adjusted *p* < 0.001) and then rebounded from during- to post-pandemic (Holm-adjusted *p* < 0.001), resulting in higher post- than pre-pandemic performance overall (Holm-adjusted *p* < 0.001) (Supplementary Table S2; Figure 2B). School type exerted a strong influence on performance (*F* = 3610.66, *p* < 0.001; partial $\eta_{p}^{2}=0.057$), with general academic school students achieving substantially greater jump distances across all phases. The phase × school-type interaction reached statistical significance but was small in magnitude (partial $\eta_{p}^{2}=0.002$), indicating that phase-related changes were modest relative to persistent between-track differences.

Overall, strength- and power-related fitness components showed no evidence of sustained post-pandemic deterioration, while educational track was associated with stable and quantitatively meaningful differences in performance.

### Endurance- and flexibility-related fitness outcomes

Endurance- and flexibility-related fitness outcomes, including forced vital capacity, endurance running speed, and sit-and-reach performance, exhibited more heterogeneous and school type dependent patterns across pandemic phases (Figure 3). Omnibus OLS tests indicated significant main effects of pandemic phase and school type for all three outcomes (all *p* < 0.001; Table 2). Forced vital capacity demonstrated clear temporal variation across pandemic phases. In general academic schools, forced vital capacity increased from the pre- to during-pandemic phase (Holm-adjusted *p* < 0.001) and then decreased slightly from during- to post-pandemic (Holm-adjusted *p* < 0.001), while remaining higher post- than pre-pandemic overall (Holm-adjusted *p* < 0.001) (Supplementary Table S2; Figure 3A). In vocational schools, the pre- to during-pandemic change was not significant (Holm-adjusted *p* = 0.92), whereas a significant increase was observed from during- to post-pandemic (Holm-adjusted *p* < 0.001), resulting in higher post- than pre-pandemic values overall (Holm-adjusted *p* < 0.001). School type was strongly associated with forced vital capacity (*F* = 5108.05, *p* < 0.001; partial $\eta_{p}^{2}=0.079$), while the phase × school-type interaction, although statistically significant, was small (partial $\eta_{p}^{2}=0.004$), indicating differences mainly in timing rather than the magnitude of change.

Endurance running speed showed a statistically significant but comparatively small main effect of pandemic phase (*F* = 11.86, *p* < 0.001; Table 2). In general academic schools, the pre- to during-pandemic difference was not significant (Holm-adjusted *p* = 0.074), whereas post-pandemic performance was higher than during-pandemic (Holm-adjusted *p* < 0.001); the post- vs pre-pandemic contrast was not significant (Holm-adjusted *p* = 0.081) (Supplementary Table S2; Figure 3B). In vocational schools, the pre- to during-pandemic change was not significant (Holm-adjusted *p* = 0.21); the during- to post-pandemic contrast was marginal (Holm-adjusted *p* = 0.052), while post-pandemic performance was higher than pre-pandemic (Holm-adjusted *p* < 0.001). School type exerted the strongest influence observed among all fitness outcomes (*F* = 6703.12, *p* < 0.001; partial $\eta_{p}^{2}=0.101$), with general academic school students consistently outperforming vocational school students across all phases. The phase × school-type interaction was statistically significant but small (partial $\eta_{p}^{2}=0.002$), indicating persistent between-track differences with only modest phase-dependent variation.

Sit-and-reach flexibility increased across pandemic phases in both school types, with small but statistically significant improvements in general academic schools (Holm-adjusted *p* = 0.036 for during vs pre; Holm-adjusted *p* = 0.040 for post vs during; Holm-adjusted *p* < 0.001 for post vs pre) and larger improvements in vocational schools (all Holm-adjusted *p* < 0.001) (Supplementary Table S2; Figure 3C). Both pandemic phase and school type showed strong main effects (*F* = 745.69 and *F* = 3201.59, respectively; all *p* < 0.001), with general academic school students demonstrating greater flexibility across phases. The phase × school-type interaction was statistically significant and of small-to-moderate magnitude (partial $\eta_{p}^{2}=0.013$), reflecting a steeper improvement trajectory among vocational school students.

Taken together, endurance- and flexibility-related outcomes displayed greater temporal complexity than strength- and power-related measures, with modest phase-dependent changes and delayed improvements in some outcomes, alongside large and persistent school-type disparities, especially for endurance performance.

## Discussion

Using large-scale school-based fitness surveillance data collected before, during, and after the COVID-19 pandemic, this study provides a phase-based assessment of changes in anthropometric and physical fitness outcomes among Chinese upper-secondary school students, with a particular focus on differences between general academic and vocational education tracks within the same surveillance system. Across the overall sample, significant phase-related variation was observed for all measured outcomes, indicating that the pandemic period coincided with measurable shifts in population-level anthropometric and physical fitness patterns. However, these changes were not uniform across the educational tracks. Statistically significant interaction effects between the pandemic phase and school type were identified across outcomes, suggesting that students attending general academic and vocational schools exhibited distinct phase-associated population-level patterns across the pandemic and post-pandemic periods, with differences in magnitude or timing. Although the Phase and School Type interactions were statistically significant, their effect sizes were generally small; therefore, practical significance should be interpreted cautiously.

First, consistent with the majority of previous studies examining the impact of the COVID-19 pandemic on youth physical fitness (16, 28, 29), our results indicate that anthropometric indicators (height, body mass, and BMI) and performance measures related to explosive power and endurance were affected among Chinese high school students aged approximately 15–18 years. However, a closer examination of phase-wise patterns suggests that the observed increases in body mass and BMI among students in regular schools should be interpreted cautiously and may partly reflect the long-term secular upward trajectory. Instead, these increases appear to align with the long-term upward trajectory of anthropometric indicators that have been consistently reported among Chinese adolescents over the past decades (13, 30). From this perspective, the pandemic did not substantially disrupt the pre-existing secular trends in body mass and BMI within the RS population. In contrast, mean height among vocational-school students did not show a clear increase from the pre-pandemic to the during-pandemic phase in our repeated cross-sectional samples. This divergence suggests that vocational-school students may have been more affected during the pandemic period. One possible explanation is unmeasured differences in activity opportunities and routines and other lifestyle factors during the pandemic; however, these mechanisms cannot be tested in our data due to the lack of physical activity and contextual measures. Taken together, these findings imply that while overall anthropometric growth trends among Chinese adolescents may be relatively resilient to large-scale societal disruptions, such resilience is not uniformly distributed across school types, with students in vocational education appearing more sensitive to external shocks, such as the COVID-19 pandemic.

Regarding explosive power, phase-wise comparisons indicated that the COVID-19 pandemic was associated with changes in adolescents' motor performance, consistent with previous reports of pandemic-related fluctuations in youth physical fitness (31–33). Standing long jump performance improved across phases in both school types, suggesting no sustained deterioration during the pandemic period in this outcome, while persistent between-track differences remained substantial. This pattern suggests that explosive performance did not show sustained deterioration across phases and may recover as activity opportunities normalize; however, the role of structured physical education and habitual physical activity cannot be directly evaluated in our data (34, 35). Between-school comparisons further highlight the persistent influence of school types. Across all phases, RS students consistently outperformed their VS counterparts in the standing long jump, with large and highly significant differences observed before, during, and after the pandemic (Supplementary Table S3). The stability of these gaps implies that structural factors associated with the educational context may exert a stronger and more enduring influence on explosive power development than short-term pandemic-related disruptions, in line with previous evidence linking school type to systematic differences in adolescent physical fitness (36). Trajectory patterns provide additional insight into this disparity: RS students exhibited a generally increasing trend in long jump performance across phases, indicating relative robustness despite pandemic-related disruption, whereas VS students exhibited a flatter improvement slope (smaller phase-to-phase gains) relative to RS, consistent with the statistically significant but small Phase × SchoolType interaction. Together, these divergent trajectories suggest that explosive power development among VS students may be more vulnerable to external disturbances, consistent with earlier studies highlighting fitness disadvantages and activity constraints among students in vocational education settings (36, 37).

In contrast to explosive power, endurance- and flexibility-related fitness outcomes showed more pronounced and heterogeneous phase-related changes during the pandemic. Importantly, these changes did not exhibit a uniform decline–recovery pattern.

For forced vital capacity, students in regular schools (RS) exhibited an increase from the pre-pandemic to the pandemic phase, followed by a partial decline thereafter, whereas vocational school (VS) students showed relatively stable values during the pandemic and a clearer increase in the post-pandemic phase (Figure 3A; Supplementary Table S2). A similar pattern was observed for endurance running: RS students demonstrated a rapid improvement after the pandemic, whereas VS students showed a more gradual increase relative to pre-pandemic levels (Figure 3B; Supplementary Table S2). Taken together, the forced vital capacity and endurance patterns are compatible with potential differences in unmeasured activity opportunities, routines, and PE exposure between school types. However, these mechanisms cannot be tested in our data because physical activity and PE exposure were not measured. Despite these school-type-specific nuances, endurance-related outcomes were characterized by substantial phase effects, indicating sensitivity to prolonged disruptions in habitual physical activity and structured physical education (31, 32, 38). This interpretation is consistent with the detraining literature showing that endurance adaptations may emerge with a delay and that recovery trajectories do not necessarily mirror the initial disruption (39, 40). Flexibility displayed a different, yet informative, pattern. Although RS students outperformed VS students in the sit-and-reach test before the pandemic, the between-school gap narrowed during and after the pandemic (Figure 3C; Supplementary Table S3). This convergence is compatible with evidence that COVID-19–related restrictions can disproportionately reduce training exposure among individuals with higher baseline activity levels, thereby temporarily compressing between-group differences in flexibility (37, 41).

## Limitations

This study has several limitations. First, phase-based comparisons were derived from repeated cross-sectional data rather than true longitudinal tracking. While this design allows for the identification of population-level temporal patterns and subgroup differences, it limits causal inferences regarding individual developmental pathways and adaptive responses to pandemic-related disruptions. Key behavioral and contextual factors, such as physical activity volume and intensity, training modalities, home-based exercise, and psychosocial stress, were not directly assessed. Second, the post-pandemic period in our dataset is limited to 2023–2024, which restricts inferences about longer-term recovery or adaptation beyond this window. Third, we did not explicitly model the clustered structure of students nested within schools; therefore, standard errors may be underestimated and p values should be interpreted cautiously. Fourth, the data were collected from two cities within a China-specific academic–vocational tracking system, which may limit generalizability to other regions or educational contexts. Finally, outcomes were derived from routine school-based assessments rather than research-grade clinical measurements, and measurement precision may vary across testing conditions. The absence of these measures may partly account for the heterogeneous responses observed across fitness domains and school types, including delayed changes in endurance performance and convergence patterns in flexibility. Despite these limitations, the consistent interaction between the pandemic phase and school type across outcomes indicates that the pandemic did not affect adolescent fitness uniformly but rather reshaped developmental trajectories in a school-type-specific manner. This finding underscores the importance of the educational context when interpreting large-scale environmental shocks. These limitations also indicate directions for future research.

## Conclusion and implications

Using large-scale school-based fitness surveillance data spanning the pre-pandemic, pandemic, and post-pandemic periods, this study provides a comprehensive overview of how adolescent anthropometric and physical fitness outcomes in China have varied across pandemic phases. The results indicate that pandemic-related disruptions were associated with measurable changes in multiple fitness domains, but these changes were domain-specific and shaped by the educational track. Anthropometric indicators showed relative stability at the population level, although students in vocational education appeared to be more sensitive to external disruptions. In contrast, explosive power was more consistently differentiated by school type than by pandemic phase, whereas endurance- and flexibility-related outcomes were more strongly influenced by temporal disruptions affecting physical activity opportunities. Taken together, these findings suggest that large-scale societal shocks do not affect adolescent physical fitness uniformly but instead reshape developmental trajectories in domain- and context-specific ways. This heterogeneity underscores the importance of fitness monitoring and intervention strategies that are sensitive to both the fitness domain and the educational context. Future research integrating multivariate and predictive approaches may further clarify the patterns of resilience and vulnerability and help inform more targeted health promotion efforts among adolescents.

Our findings suggest that post-pandemic school fitness strategies may benefit from a track-sensitive approach. First, educational track (academic vs. vocational) could be used as a stratification dimension when safeguarding minimum opportunities for physical education and structured activity during periods of disruption. Second, given that endurance and flexibility exhibited more pronounced phase-dependent changes, whereas explosive power showed more persistent between-track differences, recovery-oriented programming may prioritize endurance- and flexibility-focused activities delivered through short and frequent modules tailored to each track's time constraints and daily routines. Third, routine adolescent fitness surveillance should consider reporting and monitoring key outcomes stratified by educational track to better identify heterogeneous trajectories and inform targeted resource allocation aimed at reducing health inequalities. These implications should be interpreted cautiously because the present study is based on repeated cross-sectional data and lacks direct measures of physical activity and other contextual factors; thus, future intervention and policy evaluation studies are warranted. These results should be interpreted as average phase-associated differences rather than as definitive evidence of long-term consequences.

## Data Availability

The datasets analyzed in this study are not publicly available due to data protection and privacy regulations governing school-based administrative records. However, de-identified data may be made available from the corresponding author upon reasonable request and with permission from the data-holding authority.
