# Supplementary material for: Phase-specific changes in anthropometric and physical fitness outcomes among Chinese upper-secondary students before, during, and after the COVID-19 pandemic: the moderating role of educational track
Source: Front Public Health. 2026 Mar 4;14:1771290. doi: 10.3389/fpubh.2026.1771290 (PMC12996040; doi:10.3389/fpubh.2026.1771290)
Supplement: Supplementary file 1 [file Data_Sheet_1.pdf]

Supplementary Material

1 SUPPLEMENTARY DATA

Supplementary Table S1 presents descriptive statistics for anthropometric and physical fitness outcomes, stratified by pandemic phase and school type.

Supplementary Table S2 reports phase-wise planned contrasts within each school type for all outcomes, derived from the pooled OLS linear model (Phase, SchoolType, and Phase × SchoolType) using estimated marginal means (EMMs) with Holm-adjusted p values.

Supplementary Table S3 summarizes school-type differences within each pandemic phase (Vocational - Regular) for all outcomes, derived from EMM contrasts with Holm-adjusted p values.

Table S1. Descriptive statistics (mean ± SD) by pandemic phase and school type.

Outcome	School type	Pre		During		Post	
		N	Mean ± SD	N	Mean ± SD	N	Mean ± SD
BMI	Regular Schools	14757	20.611 ± 3.053	7085	21.010 ± 3.244	10642	21.149 ± 3.259
	Vocational schools	9735	20.700 ± 3.719	6563	21.665 ± 4.615	10656	21.434 ± 3.728
Height (cm)	Regular Schools	14757	166.737 ± 7.993	7085	167.497 ± 8.147	10642	168.087 ± 8.157
	Vocational schools	9735	167.029 ± 8.086	6563	166.799 ± 8.493	10656	169.661 ± 8.373
Standing long jump (cm)	Regular Schools	14757	198.440 ± 33.132	7085	201.493 ± 33.541	10642	205.871 ± 34.739
	Vocational schools	9735	199.990 ± 32.262	6563	196.576 ± 32.786	10656	203.006 ± 33.824
Endurance running speed (m/s)	Regular Schools	14757	3.986 ± 0.558	7085	3.968 ± 0.561	10642	4.002 ± 0.593
	Vocational schools	9735	3.785 ± 0.546	6563	3.800 ± 0.564	10656	3.821 ± 0.614
Sit-and-reach (cm)	Regular Schools	14757	14.955 ± 6.778	7085	15.203 ± 7.046	10642	15.462 ± 7.116
	Vocational schools	9735	9.326 ± 6.860	6563	10.995 ± 6.995	10656	13.732 ± 6.291
Sprint speed (m/s)	Regular Schools	14757	6.118 ± 0.710	7085	6.160 ± 0.715	10642	6.232 ± 0.741
	Vocational schools	9735	6.116 ± 0.721	6563	6.117 ± 0.748	10656	6.203 ± 0.677
Force Vital capacity (mL)	Regular Schools	14757	3824.215 ± 934.070	7085	3979.397 ± 1053.716	10642	3909.107 ± 1041.375
	Vocational schools	9735	3520.532 ± 1020.402	6563	3514.409 ± 1028.618	10656	3653.108 ± 888.067
Weight (kg)	Regular Schools	14757	57.500 ± 10.726	7085	59.145 ± 11.294	10642	59.972 ± 11.493
	Vocational schools	9735	57.919 ± 12.168	6563	60.414 ± 14.199	10656	61.955 ± 12.780

Table S2. Phase-wise pairwise comparisons within each school type for all outcomes.

Outcome	School type	During vs Pre			Post vs During			Post vs Pre		
		Mean diff	Adj. p	95% CI	Mean diff	Adj. p	95% CI	Mean diff	Adj. p	95% CI
BMI	Regular Schools	-0.399	$<$0.001	[-0.506, -0.292]	0.139	0.011	[0.025, 0.253]	-0.538	$<$0.001	[-0.632, -0.444]
	Vocational schools	-0.965	$<$0.001	[-1.113, -0.817]	-0.231	$<$0.001	[-0.377, -0.086]	-0.734	$<$0.001	[-0.864, -0.604]
Height (cm)	Regular Schools	-0.759	$<$0.001	[-1.033, -0.486]	0.591	$<$0.001	[0.300, 0.881]	-1.350	$<$0.001	[-1.591, -1.109]
	Vocational schools	0.229	0.19	[-0.081, 0.540]	2.862	$<$0.001	[2.557, 3.167]	-2.632	$<$0.001	[-2.905, -2.360]
Standing long jump (cm)	Regular Schools	-3.053	$<$0.001	[-4.197, -1.910]	4.378	$<$0.001	[3.165, 5.591]	-7.431	$<$0.001	[-8.438, -6.425]
	Vocational schools	3.414	$<$0.001	[2.179, 4.650]	6.430	$<$0.001	[5.216, 7.644]	-3.016	$<$0.001	[-4.101, -1.931]
Endurance running speed (m/s)	Regular Schools	0.018	0.074	[-0.001, 0.037]	0.034	$<$0.001	[0.013, 0.054]	-0.016	0.081	[-0.033, 0.001]
	Vocational schools	-0.016	0.21	[-0.037, 0.006]	0.021	0.052	[-0.000, 0.042]	-0.037	$<$0.001	[-0.056, -0.018]
Sit-and-reach (cm)	Regular Schools	-0.248	0.036	[-0.483, -0.013]	0.259	0.040	[0.010, 0.509]	-0.507	$<$0.001	[-0.714, -0.300]
	Vocational schools	-1.669	$<$0.001	[-1.918, -1.419]	2.737	$<$0.001	[2.492, 2.983]	-4.406	$<$0.001	[-4.625, -4.186]
Sprint speed (m/s)	Regular Schools	-0.042	$<$0.001	[-0.067, -0.018]	0.072	$<$0.001	[0.046, 0.098]	-0.115	$<$0.001	[-0.136, -0.093]
	Vocational schools	-0.002	0.99	[-0.028, 0.025]	0.085	$<$0.001	[0.059, 0.111]	-0.087	$<$0.001	[-0.110, -0.063]
Force Vital capacity (mL)	Regular Schools	-155.182	$<$0.001	[-188.954, -121.411]	-70.290	$<$0.001	[-106.117, -34.464]	-84.892	$<$0.001	[-114.606, -55.177]
	Vocational schools	6.123	0.92	[-30.276, 42.523]	138.699	$<$0.001	[102.938, 174.460]	-132.576	$<$0.001	[-164.528, -100.623]
Weight (kg)	Regular Schools	-1.646	$<$0.001	[-2.022, -1.269]	0.827	$<$0.001	[0.427, 1.226]	-2.472	$<$0.001	[-2.803, -2.141]
	Vocational schools	-2.495	$<$0.001	[-2.979, -2.011]	1.540	$<$0.001	[1.065, 2.016]	-4.035	$<$0.001	[-4.460, -3.611]

Table S3. Between-school differences within each pandemic phase for all outcomes.

Outcome	Phase	N_{Reg}	N_{Voc}	Mean diff	95% CI	Adj. p
BMI	Pre	14757	9735	0.089	[-0.000, 0.178]	0.050
	During	7085	6563	0.655	[0.520, 0.790]	<0.001
	Post	10642	10656	0.285	[0.191, 0.379]	<0.001
Height (cm)	Pre	14757	9735	0.292	[0.086, 0.498]	0.006
	During	7085	6563	-0.697	[-0.977, -0.418]	<0.001
	Post	10642	10656	1.574	[1.352, 1.796]	<0.001
Standing long jump (cm)	Pre	14757	9735	1.550	[0.716, 2.385]	<0.001
	During	7085	6563	-4.917	[-6.030, -3.804]	<0.001
	Post	10642	10656	-2.865	[-3.786, -1.944]	<0.001
Endurance running speed (m/s)	Pre	14757	9735	-0.202	[-0.216, -0.187]	<0.001
	During	7085	6563	-0.168	[-0.187, -0.149]	<0.001
	Post	10642	10656	-0.180	[-0.197, -0.164]	<0.001
Sit-and-reach (cm)	Pre	14757	9735	-5.628	[-5.803, -5.454]	<0.001
	During	7085	6563	-4.208	[-4.443, -3.972]	<0.001
	Post	10642	10656	-1.730	[-1.910, -1.549]	<0.001
Sprint speed (m/s)	Pre	14757	9735	-0.002	[-0.020, 0.017]	0.85
	During	7085	6563	-0.042	[-0.067, -0.018]	0.002
	Post	10642	10656	-0.029	[-0.049, -0.010]	0.004
Force Vital capacity (mL)	Pre	14757	9735	-303.683	[-328.943, -278.423]	<0.001
	During	7085	6563	-464.988	[-499.939, -430.038]	<0.001
	Post	10642	10656	-255.999	[-281.996, -230.002]	<0.001
Weight (kg)	Pre	14757	9735	0.420	[0.123, 0.717]	0.006
	During	7085	6563	1.269	[0.837, 1.702]	<0.001
	Post	10642	10656	1.983	[1.657, 2.310]	<0.001
